# Face/Off: Changing the face of movies with deepfakes

**DOI:** 10.1371/journal.pone.0287503

**Published:** 2023-07-06

**Authors:** Gillian Murphy, Didier Ching, John Twomey, Conor Linehan

**Affiliations:** 1 School of Applied Psychology, University College Cork, Cork, Ireland; 2 Lero, The Science Foundation Ireland Centre for Software Research, Limerick, Ireland; Kitami Institute of Technology, JAPAN

## Abstract

There are growing concerns about the potential for deepfake technology to spread misinformation and distort memories, though many also highlight creative applications such as recasting movies using other actors, or younger versions of the same actor. In the current mixed-methods study, we presented participants (N = 436) with deepfake videos of fictitious movie remakes (such as Will Smith staring as Neo in The Matrix). We observed an average false memory rate of 49%, with many participants remembering the fake remake as better than the original film. However, deepfakes were no more effective than simple text descriptions at distorting memory. Though our findings suggest that deepfake technology is not uniquely placed to distort movie memories, our qualitative data suggested most participants were uncomfortable with deepfake recasting. Common concerns were disrespecting artistic integrity, disrupting the shared social experience of films, and a discomfort at the control and options this technology would afford.

## 1 Introduction

Deepfakes are a type of manipulated media created using artificial intelligence technologies [1]. Deepfakes most often consist of videos where an artificial face has been superimposed onto another person’s face, resulting in a highly convincing recording of someone doing or saying something that they never did [2]. While computer-generated imagery is not a new technical development, the rise of deepfakes and the development of deepfake creation apps promises an especially realistic product without significant training or expensive equipment [3], and research has shown that most individuals cannot reliably distinguish AI generated faces from real faces [4]. Almost as soon as deepfake technology emerged on Reddit in 2017, discussions around the grave harm they could cause was widespread [5]. Indeed, the first deepfakes posted online were fabricated pornographic videos featuring celebrities, and non-consensual deepfake pornography remains a pressing issue [6] As well as this, there are concerns that widespread use of deepfake technology could give rise to a society where individuals lose faith in the veracity of media [7] and where video evidence cannot be used in court cases [8,9]. It has been argued that deepfakes may be especially potent at distorting memories, giving rise to false memories for events that never occurred [10,11].

More recently, the positive applications of deepfakes have been discussed. Deepfakes allow for quick and relatively cheap translation of films, marketing material and educational resources into other languages, as well as potentially restoring speech to individuals who have lost their voice to disease or injury [7,8]. For example, deepfake technology was used to depict the footballer David Beckham “speaking” nine languages in a campaign against malaria [12] and to protect the identity of LGBT individuals speaking out about persecution in Russia [13]. In 2018, the speech synthesis company CereProc used deepfake technology to produce audio of John F. Kennedy delivering the speech he was due to give on the day he was assassinated [14]. Some have suggested that deepfake technology may soon allow us to hyper-personalise our shopping and entertainment–using virtual deepfakes of ourselves to try on a pair of sunglasses before purchasing, or inserting our own faces onto video game characters [3].

One field which could be revolutionised by deepfake technology is the creative arts, especially television and cinema. It has been suggested that deepfakes could be used to translate films, edit misspoken words in a recorded scene, or to put the leading actor’s face on the body of a stunt-double [3]. Current estimates are that deepfake videos can be produced at less than ten percent of the typical cost of other synthetic media [12]. We are seeing the adoption of this technology already, with deepfakes being used creatively to improve storytelling. For example, in the Star Wars TV show “The Book of Boba Fett”, the character of Luke Skywalker is played by an actor with a younger version of the original Luke Skywalker (actor Mark Hamill) superimposed onto their face [15]. This technology has been embraced by art museums and galleries too. Painter Salvador Dalí was recreated using deepfake technology in the Dali Museum in Florida [16]. Projector screens showed Dalí move, speak, and interact with museum visitors, even inviting audience members to take a selfie with him. An exhibition in the International Centre of Photography in New York City used deepfake technology to recast the film *The Warriors*, inserting visitors into key scenes in the film [17]. In less official capacities, in the homes of movie-lovers around the globe, individuals are recasting films and posting clips to Youtube. The site is awash with highly realistic face-swaps and as noted by Meskys et al. [12] these videos are made for pure fun and as a form of creative expression.

In the imagined not-too-distant future then, deepfakes may become ubiquitous and may allow us to personalise our media and entertainment. In the current work, we explore the potential issues that may arise from such a reality. Firstly, we ask, can deepfakes can distort our memories and beliefs Moreover, are deepfakes so realistic and convincing that they will distort our memories more drastically than any other current misinformation medium? This has been suggested by some authors [10] and indeed, deepfakes may harbor a situation where “seeing is believing”, distorting our shared understanding of culture and history [18]. Secondly, we ask how audiences feel about the promised possibilities of deepfakes. In what ways would the ability to personalise our entertainment be appealing or unappealing? Understanding these questions is key in designing and regulating for a future in which deepfake technology becomes more widespread.

The current paper reports a mixed-methods study concerning deepfakes in cinema. We presented participants with a series of clips from remakes of well-known films. Some of these clips were genuine remakes that were released commercially in cinemas (such as the remakes of *Aladdin* and *Carrie*), while others were deepfakes made by Youtube and Reddit users. We assessed whether participants would form false memories for these fictional remakes, and we compared the false memory rate to participants who merely read a written description of the film, in order to assess whether deepfakes are especially likely to deceive. After completing the experiment, we showed participants some deepfake clips and explained how the technology works. Participants were asked to reflect on whether they would use this recasting technology in future if it became available. We analysed their responses using discursive thematic analysis to gain greater understanding of the benefits and risks of this technology for cinemagoers. The current study is one of the first to empirically assess the potential for deepfakes in revolutionising cinema and the implications for developers, moviemakers and society are discussed.

## 2 Literature review

While the definition of deepfakes varies slightly between papers, it is generally accepted that deepfakes are audio, images, or videos which have been manipulated using AI neural networks to swap the voice or face of one person with another [19]. Generative Adversarial Networks (GAN) are usually required to create these manipulated audio, images and videos. GAN’s consist of two neural networks. Network A (generator) is fed information from a source material and produces its own “fake” version of it, and Network B (discriminator) discriminates and identifies the fakes from the generator. The generator produces the “fake” media and this and the original source material is fed into the discriminator. If the discriminator finds discrepancies between the original and the fake, then the generator learns from this discrepancy and produces better fakes. This process continues to improve the fake media until it is indistinguishable to the discriminator. The resulting synthetic media is known as a deepfake [20]. While the early deepfake materials that emerged online in 2017 showed clear signs of manipulation, they soon became more realistic and convincing due to technological advancements [21].

There are widespread concerns that deepfakes may mislead and spread misinformation, by presenting a vivid depiction of a person doing something they did not really do. These concerns continue to grow as deepfake technology becomes ever more widely-available, in the form of mobile face-swap apps, which have topped the most dowloaded charts when released [12]. Yet, others have argued that the unrelenting focus on the negative aspects of deepfakes reflects a techno-paranoia that is devoid of nuance. For example, Broinowski [22, p. 122] argued that “dystopian predictions about deepfakes side-step their socially enriching capacity to inspire, transform, and engage, and instead position the technology as harmful by design—an instrument as unequivocal in its mendacious intent as guns are unequivocal instruments of death, irrespective of the context in which it is used, or the intentions of its user”. In the current study, we assess both the potential harms and benefits of deepfakes, as they become ever more accessible to members of the public.

### 2.1 Misinformation & false memories

Decades of psychological research has demonstrated that memory is reconstructive and does not work like a video camera [23]. Our memories for what we originally experienced can be distorted by post-event information (“the misinformation effect”). Early research in this field found that after watching a recording of a car accident, participants’ recollection of the speed the cars were going was significantly affected by the wording of the question. Those asked what speed a red car was going when it ‘hit’ the other car gave lower estimates that those asked about when the red car ‘smashed’ into the other car [24]. Those who heard the word ‘smashed’ in the investigator’s question were also more likely to report seeing broken glass at the scene of the accident, though there was none. Since this ground-breaking research, many studies have demonstrated that it is possible to plant memories of entirely false events–for spilling punch all over a bride at a wedding [25], taking a hot air balloon ride as a child [26], or witnessing fabricated political events during the Irish abortion referendum [27], Brexit [28] or the US Capitol Riots [29].

The source-monitoring framework (SMF) is one way of understanding and predicting the occurrence of false memories [30]. According to the SMF, remembering is an inferential process where we attribute our mental experiences (including images and scenes) to a particular origin (something we truly witnessed, a dream, an imagined situation, etc.). Typically, we are correct in these judgements, and we use reliable indicators of veracity to establish the source of a mental event (e.g. how vivid and detailed is the scene, how plausible is the event, etc.). False memories can occur when we inadvertently and unconsciously attribute an internally generated mental experience to an incorrect source. For example, a wealth of research has demonstrated that simply imagining a fictional event can lead to significant levels of false memories for that event (“imagination inflation”;[31]). With regard to deepfakes, there are concerns that this form of hyper-realistic doctored media may bias these judgements, making viewers more susceptible to false memories. Deepfakes are incredibly convincing and can be hard to detect, and we know that even more basic forms of doctored videos have effects on memory. For example, in a 2010 study, Wade and colleagues found that participants who viewed a doctored video that showed a fellow participant cheating at a gambling task were three times more likely to sign a statement confirming they had witnessed the cheating than those who were merely told the video existed [32]. A false confession study using doctored videos also found that those who viewed a doctored video that showed themselves cheating at a task were more likely to come to believe they had in fact cheated [33]. From a source-monitoring perspective, viewing doctored videos may increase familiarity with a fake event and increase the ease with which the event can be pictured. This can make the event seem more credible and the ease with which such a ‘memory’ can come to mind may lead participants to falsely believe it truly occurred [33]. This has led some to conclude that deepfakes are likely to be especially potent in distorting our memories of the past [10].

There is limited research on doctored video effects on memory, perhaps in part because the technical skills and equipment required to create convincing materials for such a study may not often be found in psychology research labs. This appears to be changing with the rise of simple deepfake creation apps, however we do not, as yet, have firm evidence for the effects of deepfakes on memory. A recent study compared false memories for fabricated news stories depicted as deepfakes against the same fake stories presented as either text or text alongside a photograph [34]. Though participants had negative perceptions of deepfakes after taking part in the study (rating them as dangerous, unethical and highly convincing) and many participants reported false memories for fabricated events after viewing a related deepfake, deepfakes did not reliably increase the incidence of false memories relative to less technologically advanced mediums. Thus, while the literature around deepfakes frequently cites the spread of misinformation and memory distortion as a likely harm [10], the empirical evidence to support such a claim is not yet apparent.

### 2.2 Democratising deepfakes

Deepfake technology has spread rapidly from a dedicated community on Reddit with significant technical skills, to become accesible to an average internet user with no specific training or equipment [35]. This democratisation of deepfake technology has had measurable harms for many individuals. In a classifying exercise, cyber-security company Deeptrace found in 2019 that 96% of all deepfake videos on the internet were of a pornographic nature [5]. Almost all of these were created nonconsenually and the majority, but not all, featured celebrities. With the advances in deepfake technology, a wealth of material is no longer required to train the AI and many internet users are using deepfake apps to create fake pornographic footage of those they know personally [6,13]. This has been classed as a form of technology-facilitated image-based abuse [36].

Beyond pornography, accessible deepfake technology may soon allow members of the public to regularly create and consume deepfake media. Many of these applications are creative, educational and playful, yet they do not receive the same attention in the literature as the more negative elements [22]). It has been speculated that deepfakes can and will be used to customise and personalise movies, video games, and other media by superimposing one’s face onto the characters [37]. Deepfakes are also beginning to be used by creative artists. The rapper Kendrick Lamar released a music video in 2022 (*‘The Heart Part 5’*) depicting himself rapping but superimposing different influential black men onto his face using deepfake technology. A trailer for the 2019 film *Gemini Man* starring Will Smith used deepfake technology to depict an older clip of Smith, from the show *The Fresh Prince of Bel Air*, speaking about the film. These are examples of deepfakes being used with the full awareness and consent of the stars, but popular media has featured much speculation as to how this technology will transform the film industry. A 2019 article in Collider predicted “Within five years, deepfakes will have advanced to the point that it will not only be possible but probable we’ll be watching the likes of Marilyn Monroe co-starring opposite Heath Ledger in entirely original feature films” [38]. Others have speculated that the option to recast oneself in popular media will soon be extended to members of the public; “it is inevitable that, quite possibly by the time you read this article, the technology will be available for anyone to ‘star’ not only in high quality deepfakes of scenes from TV shows, but also in any commercial they want” [3, p. 476]. Despite these technological advances opening up new possibilities for public participation in films, little is known about how we should design these digital tools or how we would limit any harms they may cause.

### 2.3 Research gap

Existing research has highlighted the potential for misinformation to distort memory [23] and the emergence of deepfakes has heightened these concerns [10]. However, the positive and creative applications of deepfakes are also an exciting area of speculation, one that promises to offer new possibilities and a world of choice to members of the public [3]. In the current study, we sought to answer two research questions to better understand how deepfakes may affect cinema in the near future: Q1. Do deepfake clips increase false memories for fabricated films? Due to a lack of empirical deepfake research, we do not yet know if deepfakes are uniquely poised to distort memories and the current study will assess this in regards to memories of films. Q2. Why would moviegoers choose to use this technology, or not? Though it is clear that the possibilities of this technology are expanding, we do not yet understand what would make cinematic applications attractive to members of the public. What features ought to be considered when designing this technology and what harms should we plan to mitigate? The current study will thus advance our understanding of deepfakes in the film industry.

## 3 Method

### 3.1 Method

This study was preregistered (available here https://aspredicted.org/blind.php?x=RHM_5BB). The materials and data are available at https://osf.io/czy9v/.

### 3.2 Participants

Participants were recruited by undergraduate students as part of a class project. 570 participants completed the survey in full, however, in line with our preregistration we removed those who reported searching the internet or asking for help to answer the questions (n = 20) or reported not watching the videos (n = 111). We also removed three participants who were under 18. This resulted in a final sample of 436 participants. Most (n = 253) were female, 173 were male, 3 participants reported their gender as non-binary, and 7 preferred not to say. The sample had an average age of 25 (SD = 20, range 18–65). Just over a third of participants (35%) had completed an undergraduate or postgraduate degree. Participants varied in their interest in film; when asked whether they considered themselves “movie buffs”, i.e. an expert in films who watches films regularly and keeps up with new releases, participants responded that this describes them perfectly (5%), this sort of describes them (30%), they weren’t sure (11%), this doesn’t really describe them (38%), or this doesn’t describe them at all (16%).

### 3.3 Materials & procedure

Participants completed an online survey where they were presented with real and fake movie remakes. The study was described as investigating opinions about recent movie remakes. Participants first provided some demographic information before being asked to complete an auditory test to check their speakers were working. Participants were then presented with six films in a random order (four true and two fake). Three of these films were presented as a short text description and three were also accompanied by a short film clip.

The four fake movie remakes presented in the study were The Shining, The Matrix, Indiana Jones, and Captain Marvel. The text descriptions were: “In 2012, Brad Pitt & Angelina Jolie starred in a remake of The Shining. The real-life couple played Jack & Wendy Torrance in the Stephen King horror film.”, “In 2008, Will Smith starred in a remake of The Matrix. Smith played Neo in the film about a dystopian future in which citizens are trapped inside a simulation.”, “In 2013, Chris Pratt starred in a remake of Indiana Jones. Pratt played the titular archaeologist Jones, who journeys to seize the Ark of the Covenant.”, “In 2020, Charlize Theron starred in a remake of Captain Marvel. Theron played the leading role in the Marvel Comics film.” The four real movie remakes presented in the study were Charlie & The Chocolate Factory, Total Recall, Carrie, and Tomb Raider. These were accompanied by similar descriptions and real clips. All clips are available in full with the study materials at the link above.

 For each film, participants were asked “Have you seen the original [film title]?” and “Have you seen this remake of [film title]?” and could respond “Yes I’ve seen the entire film”, “I’ve never seen the full film, but I’ve seen clips/trailers”, “I’ve never seen this film, but I’ve heard of it”, “I’ve never seen or heard of this film”, or “I’m not sure”. As preregistered, we coded participants who selected A, B, or C as having a memory of that film, participants who selected D were classed as having no memory of that film, and participants who selected that they were unsure were excluded from analyses.

Participants were then told that “The real purpose of this study is to examine false memories for movies. You may have been presented with some clips/film descriptions that were not in fact real remakes, but doctored videos and made-up descriptions” and asked to consider whether all the movies they had seen were real. Participants could rate each of the six films they had seen from 0 (definitely not real) to 100 (definitely real).

Next, participants were debriefed and told which of the films they saw were fake. Deepfake videos were described as following; “A deepfake video is a doctored video created using machine learning. These videos can appear highly realistic and can depict someone saying or doing something they never did.” Participants were presented with all four deepfake videos that were used in the study and were asked to watch them and rate how convincing and realistic the video was and how likely most people would be to fall for it, on a scale of 0 (not at all) to 100 (extremely).

Participants were then asked “Some people suggest that in future we could use deepfake technology to choose the actors and actresses in films to suit our personal tastes (e.g. if you didn’t like Jack Nicholson in The Shining, you could simply recast the character as Brad Pitt). Would you like to see this type of technology become available?” and could answer from Definitely Yes, Probably Yes, Might or Might Not, Probably Not, Definitely Not. Participants were then prompted to tell us why or why not, responding using an open text box. Lastly, participants were asked “If you could recast actors in films or TV series, what would motivate you to do so? (select all that apply)” and could select from “If I found the original actor annoying”, “If I disagreed with the original actor’s politics or actions and didn’t want to watch a film with them in it”, “I would use this for the novelty”, “If I preferred the replacement actor for any reason”, “If the replacement actor better fitted my perception of the character”, “If I wanted to increase the diversity of the cast”, “Other (please describe)”. Participants were then further debriefed and thanked for their participation.

### 3.4 Analysis

The quantitative data was analysed and reported as per our preregistered plans. The qualitative data was analysed following Braun and Clarke’s [39,40] guidelines for carrying out reflexive thematic analysis. Specifically, the data was first read closely. Where a response included a number of distinct ideas, it was divided into separate chunks for the purposes of open coding. Thus, 292 individual points, taken from the responses of 263 participants, were open coded. Analysis then progressed towards defining and iteratively refining a set of themes that meaningfully summarise the thoughts expressed by participants, making sure that themes always reflected the data.

### 3.5 Ethics

The study was approved by the School of Applied Psychology Ethics Committee at University College Cork. Participants provided informed consent at the start of the online survey, by clicking to indicate their consent. As misinformation researchers must ensure they are not causing harm with their experimental methods [41]), participants were debriefed using techniques that have shown to be effective in prior false memory research [42,43].

## 4 Results

### 4.1 Quantitative analysis

Participants rated the deepfakes as moderately convincing on average, on a scale of 0–100, where 100 is extremely convincing—The Shining remake (M = 61.78, SD = 31.02), The Matrix remake (M = 50.46, SD = 33.11), Captain Marvel remake (M = 70.39, SD = 31.08), Indiana Jones remake (M = 71.96, SD = 27.47). Participants gave similar answers when asked how likely others would be to fall for this video, with 100 being extremely likely—The Shining remake (M = 61.70, SD = 30.12), The Matrix remake (M = 55.66, SD = 32.76), Captain Marvel remake (M = 68.60, SD = 30.28), Indiana Jones remake (M = 67.65, SD = 28.81).

Participants readily formed false memories for these fictitious movies. Captain Marvel was most frequently falsely recalled (73%), followed by Indiana Jones (43%), The Matrix (42%), and The Shining (40%). Of those who falsely remembered each film, 41% remembered the Captain Marvel remake was better than the original, 13% remembered the Indiana Jones remake was better than the original, 12% remembered The Matrix remake was better than the original, and 9% remembered The Shining remake was better than the original.

**Fig 1:**
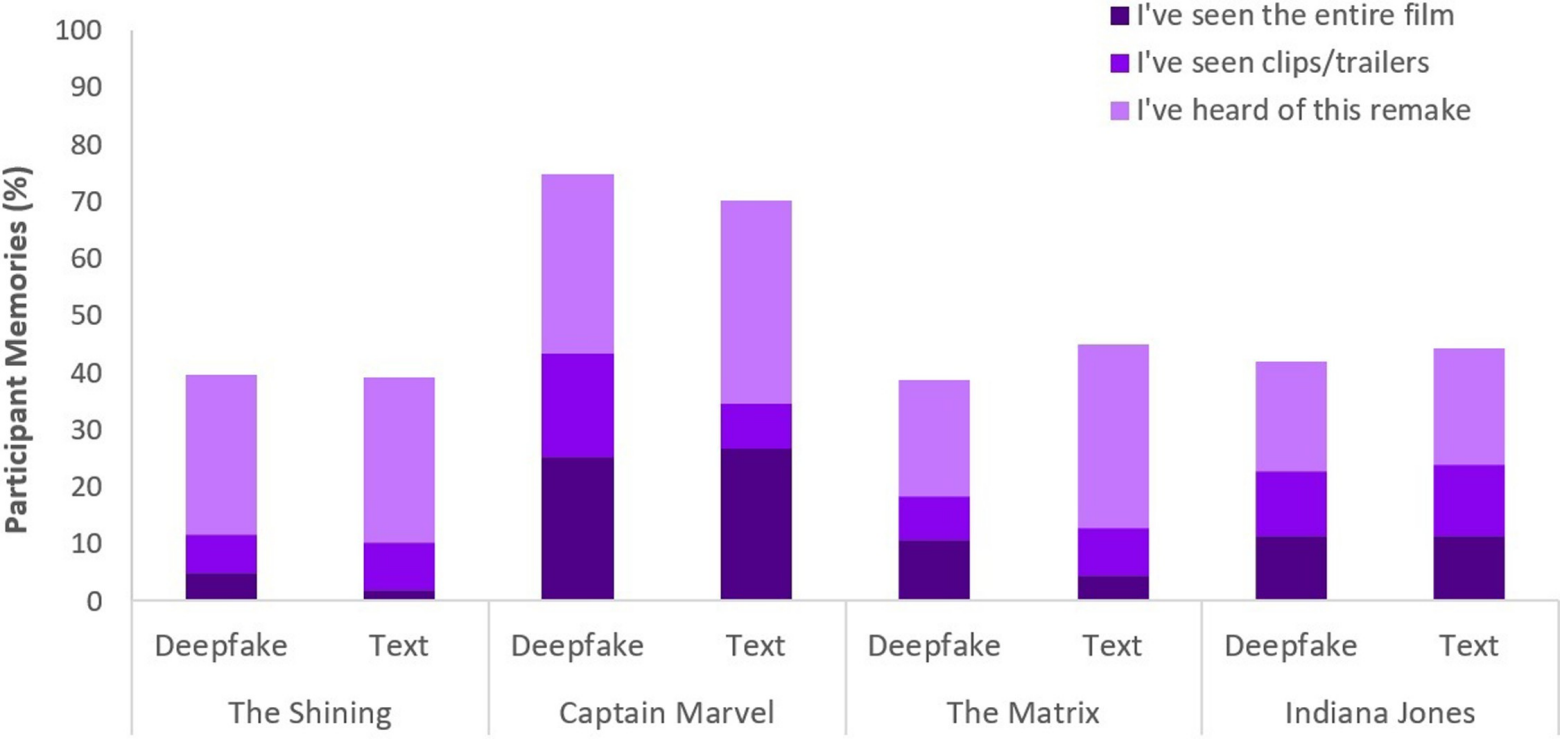
Participant memories for each of the four fictitious movie remakes.

Our primary question was whether presenting the movie in the format of a deepfake would increase the false memory rate. As shown in Fig 1, this was not the case. The Shining was remembered at a rate of 40% when presented as a deepfake and 39% when presented as text. Captain Marvel was remembered at a rate of 75% when presented as a deepfake and 70% when presented as text. The Matrix was remembered at a rate of 39% when presented as a deepfake and 45% when presented as text. Indiana Jones was remembered at a rate of 42% when shown as a deepfake and 44% when presented as text. False memory rates did not differ for any of the movies according to presentation format (all p > .05).

Interestingly, presenting participants with videos did not have a major impact on memories for the real films either. The Tomb Raider remake was remembered by 74% as a video, 64% as text, the Charlie & The Chocolate Factory remake was remembered by 97% as a video, and 98% as text, Total Recall was remembered by 49% as video and 43% as text, and Carrie was remembered by 61% as video and 55% as text.

After viewing each film and reporting their memories, participants were asked to consider whether all the films they saw were real, and to rate the truthfulness of each film on a scale of 0 (definitely not a real film) to 100 (definitely a real film). To assess whether truthfulness varied by presentation format and whether the movie was real or fake, we conducted a 2x2 repeated measures ANOVA with the factors of presentation format (text, video) and film type (real, fake). We found a significant main effect of presentation format F(1, 344) = 7.05, p = .008, ηp2 = 0.02. On average, films presented as videos were rated as more truthful (M = 49.88, SD = 21.10) than those presented as text (M = 45.89, SD = 21.47). We also found a large significant main effect of film type, F(1, 344) = 335.16, p < .001, ηp2 = 0.49, with real films rated as more truthful (M = 66.24, SD = 19.69) than fake films (M = 29.53, SD = 28.60). We also found an interaction effect between presentation format and film type (F(1, 344) = 14.15, p < .001, ηp2 = 0.04) such that the difference between truthfulness ratings for real movies presented as video (M = 70.79, SD = 25.24) and text (M = 61.70, SD = 26.78) was greater than the difference between fake movies presented as video (M = 28.97, SD = 35.59) and text (M = 30.08, SD = 34.83).

When asked whether they would like to see wider availability of this kind of technology (using deepfakes to swap actors in and out of films), participants had widely diverging responses. About a third were keen to see this technology become more widespread (12% Definitely Yes, 19% Probably Yes), 15% were unsure, and over half did not want to see this technology become more widespread (31% Definitely Not, 24% Probably Not). When asked why they would use this technology, participants selected many of our suggested answers as well as providing their own reasons. The most popular selected reason was “If the replacement actor better fitted my perception of the character” (n = 174), followed by “If I found the original actor annoying” (n = 139) and “I would use this for the novelty” (n = 121). Some participants also selected reasons related to diversity or politics: “If I disagreed with the original actor’s politics or actions and didn’t want to watch a film with them in it” (n = 96), “If I wanted to increase the diversity of the cast” (n = 80). Others simply selected “If I preferred the replacement actor for any reason” (n = 74).

### 4.2 Qualitative analysis

Our intention was to understand, describe and discuss our participants’ thoughts about the use of deepfakes in films, given all they had learned about deepfakes through participating in the study. Data describes our participants’ excitement and imagination, as well as their fears and concerns. 263 participants responded to the prompt; “Some people suggest that in future we could use deepfake technology to choose the actors and actresses in films to suit our personal tastes (e.g. if you didn’t like Jack Nicholson in The Shining, you could simply recast the character as Brad Pitt). Would you like to see this type of technology become available? Why, Why not?.” Responses varied in terms of length and depth, from short, vaguely positive responses, to lengthy discussions of art, employment and copyright law, and ethics.

**Fig 2:**
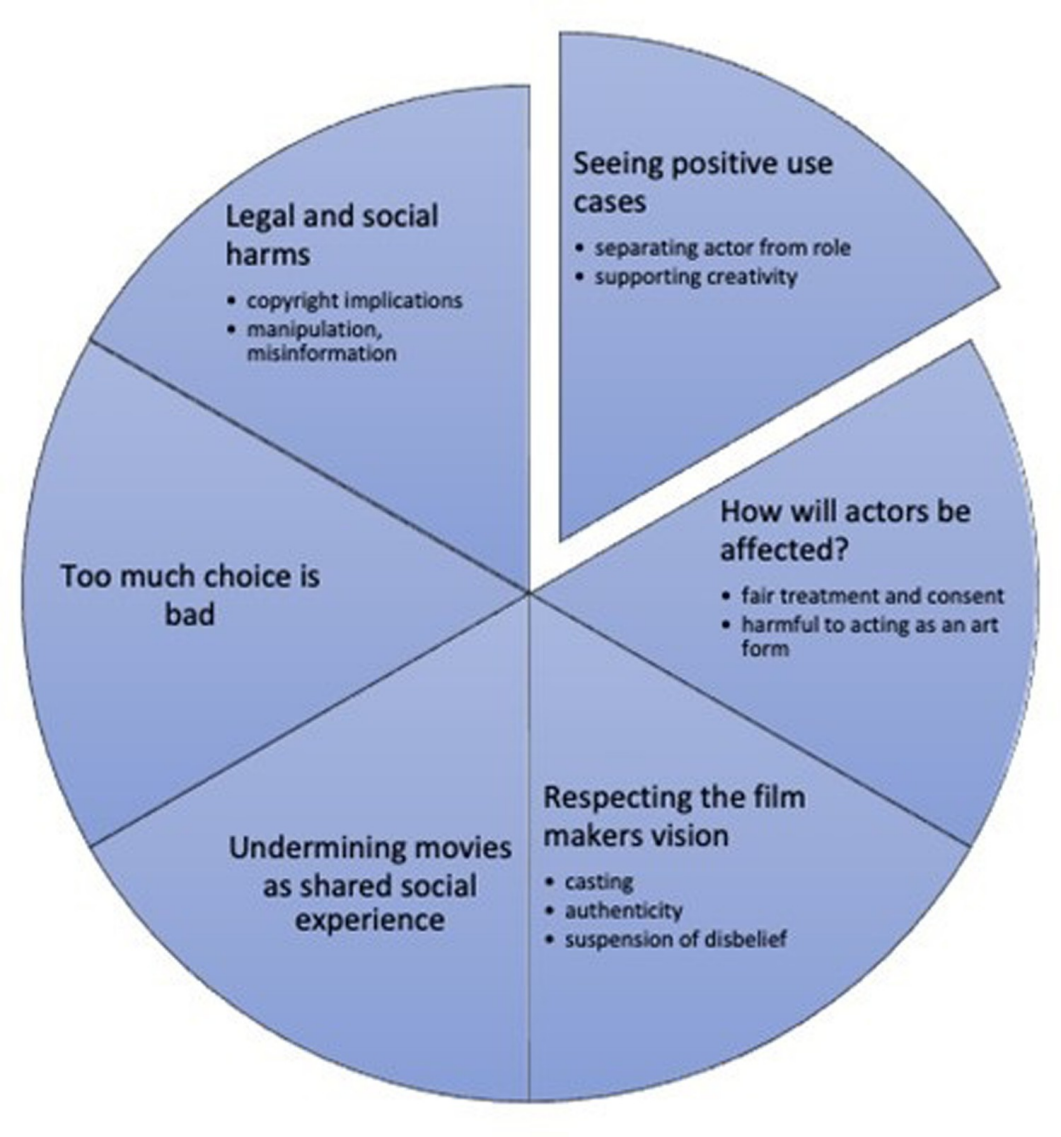
The thematic structure of participant responses.

Some brief comments expressed positive sentiments about deepfake technology being used to replace actors, but without any justification or argumentation to support that position. For example, many participants made statements such as, “It would be cool and interesting,” or, “could improve the movie.” While it is important to acknowledge that these positions exist, it is difficult to analyse such short comments in a meaningful manner. Similarly, there were many vaguely negative comments entered without any justification. Statements such as, “it’s dumb,” “unnecessary,” “little more than a novelty”, “creepy” and “it is weird and unnerving” were entered. However, the vast majority of comments were at least a full sentence in length, and provided some justification for the author’s position. Fig 2 illustrates the overall thematic structure of our findings. There are six main themes, with five focused on negative aspects and one focused on positive aspects. Four of these main themes had multiple subthemes. We will discuss each theme in turn, providing examples of participant comments described by each theme.

#### 4.2.1 Seeing positive use cases

Participants mostly demonstrated negative views when asked about the possibility of using deepfake technology to replace actors in a film. Indeed, much of the research and scholarship on deepfakes expresses similarly negative viewpoints [13,44]. However, there were some positive responses from participants also. These responses can mostly be classified into two sub-themes; separating actor from role, and supporting creativity.

Many participants expressed that the possibility of separating actors from roles could be a positive development, and two contrasting reasons were provided. Firstly, a number of participants mentioned situations where their dislike of a specific actor (e.g., “If the actor was a bad person”) impairs their enjoyment of a film. For example, “beloved movies that cast a controversial or hated actor/actress have the opportunity to recast and make the film more enjoyable.” Indeed, many participants mentioned that they do not like specific actors, and consequently do not watch their films. Interestingly, the idea of re-casting controversial actors may provide a way for people to feel they are “separating the artist from the art”, a challenge that has received renewed attention since the onset of the #MeToo movement [45]. Secondly, many participants mentioned that they have “favourite actors” who they would enjoy watching in new roles, or in some of their favourite films; “you can always have your favourite actors in whatever movie you want” and “if you like watching certain actors for certain reasons (their looks!!) I would cast them in a lot more movies.” Participants also caution that the ability to separate actor and role could be abused and could, “in some situations lead to racism (replacing POC actors with white ones).” Indeed, anywhere that power is given to audiences to participate in the generation of content, there should be some consideration given to the abuse of that power, and to mitigating actions [46].

A number of participants suggested positive uses of deepfakes that were not simply utilitarian, but could be considered creative. Indeed, participants were excited that deepfake technology could, “allow for audience participation in the creative process.” It would “be interesting and comical to change different actors into different movie parts” and “could be nice to have Nicholas Cage in every movie.” This is in line with the enormous volume of recast film clips currently available on Youtube and Reddit, contributed by average internet users, as well as the explosion of accessible and low-cost apps that allow the creation of deepfakes or less sophisticated ‘cheap fakes’ [7]). Participants also mentioned that movie makers may find uses for the technology themselves, “especially the potential for creative approaches to archive animation and the replacement of lips instead of dubbing.” There are relatively few positive examples of deepfakes, or even positive discussions of deepfakes, in the existing academic literature, with most publications focusing on the harms and risks [13,37,47]. Though the majority of our participants’ responses focus on describing harms, it is valuable to see our participants thinking about how deepfake technology can also be used in playful, creative and empowering ways.

#### 4.2.2 How will actors be affected?

Many participants chose to answer the question by speculating how deepfake technology could have harmful effects on both the profession and the art of acting. Some discussed how using deepfake technology to replace actors in films is unfair to actors; either to those who originally starred in the films, whose “hard work” and performance could now go uncredited or unacknowledged, or to an actor who had not agreed to star in a particular type of film through choice, and whose choice has now been removed, “people deserve to have their reputation protected, and their face attached to only films they consent to” “like when Galaxy [a brand of chocolate] cast a deepfake Audrey Hepburn in their commerical a few years ago” Indeed, participants frequently discussed the harms caused by famous faces being applied to pornographic movies, a common concern in the academic literature [6,36]. Participants expressed concern about how, and whether, actors would get paid in a situation where they are replaced in a film, “It depends on whether the actor whose likeness is used will be properly compensated” and, “people should be paid for their labour.” They also worried that the technology could lead to an ever decreasing pool of actors starring in films, as the same famous faces become applied to an increasingly broad range of films.

One of the most frequent points made across all participants, was a concern over how deepfaked performances could be lower in technical quality than genuine performances. Participants made the point that acting is not just a way of selling films; it is an art form; deepfakes, “will take away from the beauty of authentic, real acting.” Participants complained that the digital swapping of actors faces is likely to diminish the quality and nuance of acting in the film, “Faking these things takes the power out of the original performance. It would be nothing but a detriment to the craft”, “I think the performance of the actor or actress is more important than their face” and “some actors aren’t interchangeable, their talent is unique.” Nobody suggested that deepfaked performances could be more convincing or engaging than live recorded performances, indeed a number of participants made the point that, “there’s no way to deepfake a bad performance away.”

#### 4.2.3 Respecting the film makers’ vision

Many participants felt that the prospect of replacing actors in films using deepfake technology is ultimately unfair on the people who make movies. Participants wrote about the importance of respecting the original vision of the director and producers, and the choices they make in bringing a story to film. Again, participants pointed out that filmmaking is an art form, and that “the artistic direction of the director and production should be valued and respected” where “the director knows what they are doing better than the viewer most of the time.” Participants mentioned that the technology may be commercially successful, but would be harmful to movies as an artistic expression, “the technology would only serve to profit media conglomerates, and would disempower the artistic choices of the filmmaker”, “the loss of humanity behind art scares me” and “art should not be customisable to the viewer.” This theme has three sub-themes, all of which received significant discussion from our participants.

Many participants commented that casting decisions are intentional, and that finding the right actor for the role is a core part of what makes a film unique and memorable. “I think that casting directors know what they’re doing when they’re choosing actors to play characters” and “As much as the world would currently love a button that turns every action hero into Keanu Reeves, it’s artistically bankrupt and dismisses the work that goes into casting appropriately for movies.”

Frequently, participants suggested that the “authenticity” of a film would be harmed in a situation where actors were replaced using deepfake technology. Typically, this critique was quite vague, (e.g. “Authenticity cannot be deep faked”) and, indeed, similar points were made when referring to casting choices and the art of acting. However, given the frequency with which the word “authenticity” was used in posts, we felt this was worth identifying as a separate concern. It appears that participants are concerned with an overall trend of decreasing “realism” in films, “Too much tech. Need something to keep movies somewhat real”, “Removes the validity & originality for future generations to come. Brings in the question of ‘who/when was the original’.”

A number of participants mentioned how deepfaked versions of films may undermine the viewers’ experience of immersion in a story, or harm their experience of the magic of cinema, due to noticing “uncanny” errors with the technology, or wondering whether the version they are watching is the one intended by producers. It “sort of interrupts the fantasy aspect of the film.” One participant suggested “I like movies to escape. Not to select variables.”

#### 4.2.4 Undermining movies as a shared social experience

A number of participants focused not on how deepfakes could impact the quality of the media itself, but on how deepfakes could impact how people use and discuss that media socially. For example, one participant noted that “if everyone is experiencing a different version of the film they watch, it wouldn’t make for very good conversation or debate amongst people who watch it.” Another commented “I think part of the enjoyment of a film is seeing the same one as others and being able to talk about it.” The concern participants expressed regarding the social experience of film viewing is perhaps not the first that comes to mind when thinking of deepfakes, but it certainly reflects a branch of academic scholarship that sees media as shared cultural capital, important for building cultural ties [48]. In this regard, the erosion of film as a shared experience is certainly a harm worth considering.

#### 4.2.5 Legal and social harms

Given that much of both the academic literature and media reporting on deepfakes focuses on their potential to cause serious harm through manipulation, misinformation and personal attacks [8,13], surprisingly few of our participants focused on these issues when considering the replacement of actors in movies. However, some participants did raise concerns over deepfakes that went beyond considering their impact on movies and movie watching. These points form two subthemes, manipulation and misinformation, and copyright implications.

A number of participants made statements about how deepfakes could be used in future to misrepresent, manipulate and misinform. Participants discussed how deepfakes could be used for propaganda purposes, “imagine a place with heavy censorship using deep fake videos in their news cycle to convince the populace of things that aren’t true. Things like that make it dangerous.” They expressed concerns over how doctored videos could be used in legal proceedings to undermine the justice system, “Could lead to doctored evidence”, “everyone is at risk of having their face placed on any video/film.” Finally many argued that the technology could be used to place unknowing people into sexualised or pornographic videos without their consent, “Has dangerous repercussions re IBSA [image based sexual abuse]”. These findings suggest that many of the concerns that have dominated the literature concerning deepfake technology are also prominent in the minds of the public [6,36].

A surprisingly small number of participants cited concerns over copyright law, licensing and plagiarism. Participants questioned who would get paid for films that included re-casted actors, and how that payment process would work. In practice, actors negotiate different types of contracts for different types of films; some based on flat fees, some including profit sharing, etc. Making those calculations for each actor that may be re-cast in a role involves significant legal work, especially as copyright exemptions may apply for educational or satirical use of images [49]. For example, “you would need to license the likeness of an actor and the tech for paying those royalties does not exist yet.” Participants seemed skeptical over whether studios would endorse the deepfake re-casting of their films.

#### 4.2.6 Too much choice is bad

Interestingly, a number of participants took the opportunity to reflect more broadly on recent trends in media, where consumer choice is prioritised over artistic merit. Participants reported frequently feeling pandered to. For example, one participant complained, “why do we need everything to please us?. …. it’s a pity to see human emotion and capacity and skill being replaced by technology, mainly to ‘please’”. Another argued, “we don’t and shouldn’t always get what we want. I feel like this is another step towards a Wall-e type future.” Participants here appear to be expressing a general concern for how technological development is being directed. Deepfake movie replacement is seen by these participants as part of throw-away culture (e.g.,[50]), but more broadly as a symptom of a system designed to please rather than excite, challenge, or inspire [51].

Overall, while there certainly were a large number of comments from participants who were interested or excited about the prospect of being able to swap a favoured actors face into an existing film, the majority of comments explored various negative implications of doing so, which ranged from concerns about the quality of acting, empathy for the movie makers and their artistic vision, to thoughts about how the shared social experience could be undermined.

## 5 Discussion

As expected, participants readily formed false memories of fictitious remakes of popular films. This is in line with much false memory research [23] and demonstrates the malleability of memory when exposed to misinformation. Our primary question, though, was whether the presentation format (deepfake video vs. text) would influence false memory rates. Our data suggests that there is nothing especially potent about deepfake media when it comes to manipulating memory for cinema. This replicates prior work on deepfake fake news stories [34] and other studies on misinformation media. Many studies have demonstrated that misinformation in non-technical forms like simple narratives are extremely effective at distorting memory [25,28,52,53]. One study of autobiographical memory exposed individuals to either a doctored photograph or misleading text that suggested they had taken a hot air balloon ride as a child. They found the misleading text was actually *more* effective at distorting memory, with 80% of those in the text condition forming a false memory, compared to 50% of those who saw a doctored image of themselves in the hot air balloon [54]. This somewhat unintuitive finding may be due to the nature of memory–by providing doctored images or deepfake videos to participants, we supply such concrete information that the participant doesn’t need to do any imagining or mental construction of the event, thus they potentially have less information which they might later mistake for a real memory in a source-monitoring error [55]. Our findings suggest that this fluency-based account, which was developed in relation to autobiographical memory, may also apply to memory for cultural artifacts such as films. This should be further assessed in future research across other kinds of memory.

The implications of our findings for memory research are perhaps not as simple as suggesting we may be overestimating the memory distortion effect of deepfakes. We would note that the false memory rate for the deepfake prompts were still quite high (49% on average). Instead, we suggest that we might be underestimating how readily our memories can be distorted without any technological input. A great deal of research has demonstrated that relatively simple methods can be extremely effective at planting false memories, such as encouraging participants to imagine an event [31] or reading a headline describing an event [27]. Despite this wealth of evidence, we as researchers might still assume that doctored media would be a more effective means of distorting technology [10].

In light of this evidence, it is interesting to reflect on the alarmist discourse around deepfakes and misinformation. Some have predicted that deepfakes will bring about an ‘infocalypse’ [56]and may be particularly likely to distort our memories of the past (Liv & Greenbaum, 2020). Why have these narratives proliferated without much in the way of empirical evidence? Broinowski [22] argued that moral panics around emerging technology are nothing new–from fears that the written word would destroy human memory and that lightbulbs may cause blindness. We often project our current fears and negative experiences when we attempt to predict how we will use technology in the future [57]. It is perhaps not surprising then, in this ‘post-truth’ age of ‘infodemics’ and ‘fake news’ [58–60] that we have established a shared narrative that casts deepfakes as a threat to democracy and society. Without a doubt, deepfakes have the potential to misinform and to cause real harm, but more empirical evidence is required before we can quantify these harms, weigh the benefits, and intervene where necessary.

Our qualitative data revealed a wide variety of perspectives on the use of deepfakes in cinema. We were impressed with the depth and consideration of participant responses, with many considering the potential harms; to actors, film makers, and the art itself. There is already some scholarship predicting the not-too-distant future where deepfakes become widespread and considering the many ethical and legal implications of ‘resurrection technology’ where deceased actors are cast in films [61] though our participants raised wider issues worthy of consideration. Our participants also reflected on the nature of cinema and the potential disruptive effects of deepfake recasting, voicing concerns about losing the shared social experience of cinema and their concerns that so much choice would not be enjoyable. Positive remarks were far less frequent than negative remarks, reflecting the negative discourse in academic literature around deepfakes [22]. However, the focus on the negatives may in part have been due to the study design. By priming participants to consider the misinformation risks inherent in deepfake technology and attempting to distort their own memories for films, they may have more easily conjured negative applications rather than positives.

### 5.1 Implications

The primary implication of the current study for research is the need for more empirical investigations of deepfake technology. Though there are a great many published papers discussing potential regulation and mitigation methods for deepfakes [62], we cannot hope to mitigate against harms that we have not yet quantified. While some have suggested deepfakes may have a uniquely powerful effect on memory [10] further research is needed to explore this across different domains, assessing deepfakes covering different topics. In particular, this research should draw on existing theories and frameworks of memory distortion, such as the source-monitoring framework [30] to establish how and when deepfakes may distort memory. We also support growing calls for a greater focus on the potential positive applications of deepfakes, encouraging researchers to consider the potentially beneficial uses alongside the predicted harms [63]. As outlined by Broinowski [22], utopian deepfake predictions can be ameliorative (improving everyday life), transformative (reshaping reality) and natural (they are a logical extension of existing social systems). There is existing evidence of helpful and playful applications of deepfakes for learning (creating videos of historical figures to teach students), wellbeing (creating AI-generated fitness coaches that resemble someone you look up to—or a fitter version of yourself), and medicine (allowing dementia patients to interact with younger versions of their friends and family that they may remember) [7,64], though much of this research is speculative and descriptive rather than empirical.

Our findings also have implications for design, particularly in relation to participant concerns that the ability to recast films would offer too much choice. Indeed, even without the abundance of options offered by deepfake recasting, streaming viewers already report being paralysed by choice. The ‘paradox of choice’ reflects human tendencies to get overwhelmed by too many choices and either giving up or making poor choices [65]. Research suggests an average Netflix viewer loses interest after 60–90 seconds of searching, having reviewed 10–20 titles, after which the risk of abandoning the service increases substantially [66]. In 2021, Netflix launched a ‘Play Something’ feature, a shuffle option where Netflix will select something for you to watch. This feature allows the viewer to bypass all decision making and its very existence speaks to the pressure and paralysis that can accompany too much choice [67]. Deepfake recasting then is perhaps a risky addition to the viewing experience, as viewers would be required not only to select the film or series they would like to watch, but to cast the show as well. This has implications for the design of such features, where perhaps limiting the options would somewhat paradoxically improve the viewer experience.

### 5.2 Limitations & future research

A limitation of our false memory experiment is that the experience of viewing a deepfake clip in an online study is likely different to how participants may be exposed to deepfakes in the wild. Our participants saw the deepfake only once, while research shows that repeated exposure to misinformation typically increases belief–the illusory truth effect [68,69]. Source credibility can also affect misinformation susceptibility [70] and participants may make judgements about deepfakes encountered on social media or received from friends that may differ from those within the confines of a controlled psychology experiment. Many other factors may also play a role in susceptibility to deepfake movie remakes (such as having seen the original, how recently it was released, etc.), as previous research has suggested factors such as interest and knowledge can affect false memory susceptibility [71,72]. However, the current study represents an important step in establishing the baseline risks of memory distortion as a consequence of deepfake exposure. As noted above, while the generally negative responses in the qualitative responses to our study is similar in tone to the typical pessimistic academic publications and media coverage of deepfakes [22] the design of our study may have encouraged participants to consider the negative applications of deepfakes such as misinformation. The use of an online survey also meant that we couldn’t further interrogate participants about their responses or tease out their reasons further, though we would note that many participants gave thoughtful and considered answers reflecting complex feelings about the subject.

The current study considers deepfakes in just one domain (cinema) and we make no claims that our findings would generalise to other applications. Further research is required to understand user perspectives on the inclusion of deepfakes in education, marketing, gaming and the many other fields where the potential benefits of deepfakes have been discussed [3,7]. In addition, the effects of deepfakes on viewers’ beliefs, memories and behaviours needs to be considered across a wide variety of settings and subjects. As deepfake technology become ever more accessible, with apps that require less expertise and are cheaper than before, it is vital that we gather a broader evidence base to understand this technology which is likely to become a part of everyday life.

## 6 Conclusion

The findings of the current study suggest that with regard to cinema, deepfakes may not have the powerful effect on misleading memory that some have suggested [10]. Though deepfakes are currently being used in the creative arts and there is speculation as to how ubiquitous and democratised the technology may become [3,38], our participants expressed some reservations about such a future, while also considering the potential benefits. Deepfakes are sometimes positioned as an ‘alien technology’ and are the subject of growing social concern [63]. We support growing calls to understand deepfakes as a cultural technology, where social concerns and fears should be engaged with critically and any interventions or regulations should be evidence-based.
